# Genetic Control of Reproductive Traits in Tomatoes Under High Temperature

**DOI:** 10.3389/fpls.2020.00326

**Published:** 2020-04-24

**Authors:** Maria José Gonzalo, Yi-Cheng Li, Kai-Yi Chen, David Gil, Teresa Montoro, Inmaculada Nájera, Carlos Baixauli, Antonio Granell, Antonio José Monforte

**Affiliations:** ^1^Instituto de Biología Molecular y Celular de Plantas, Universitat Politècnica de València-Consejo Superior de Investigaciones Científicas, Valencia, Spain; ^2^Department of Agronomy, National Taiwan University, Taipei, Taiwan; ^3^Enza Zaden Centro de Investigación S.L., Almería, Spain; ^4^Centro de Experiencias de Cajamar en Paiporta, Paiporta, Spain

**Keywords:** pollen viability, fruit set, QTL, introgression line, tipburn, abiotic stress

## Abstract

Global climate change is increasing the range of temperatures that crop plants must face during their life cycle, giving negative effects to yields. In this changing scenario, understanding the genetic control of plant responses to a range of increasing temperature conditions is a prerequisite to developing cultivars with increased resilience. The current work reports the identification of Quantitative Trait Loci (QTL) involved in reproductive traits affected by temperature, such as the flower number (FLN) and fruit number (FRN) per truss and percentage of fruit set (FRS), stigma exsertion (SE), pollen viability (PV) and the incidence of the physiological disorder tipburn (TB). These traits were investigated in 168 Recombinant Inbred Lines (RIL) and 52 Introgression Lines (IL) derived from the cross between *Solanum lycopersicum* var. “MoneyMaker” and *S. pimpinellifolium* accession TO-937. Mapping populations were cultivated under increased temperature regimen conditions: T1 (25°C day/21°C night), T2 (30°C day/25°C night) and T3 (35°C day/30°C night). The increase in temperature drastically affected several reproductive traits, for example, FRS in Moneymaker was reduced between 75 and 87% at T2 and T3 when compared to T1, while several RILs showed a reduction of less than 50%. QTL analysis allowed the identification of genomic regions affecting these traits at different temperatures regimens. A total of 22 QTLs involved in reproductive traits at different temperatures were identified by multi-environmental QTL analysis and eight involved in pollen viability traits. Most QTLs were temperature specific, except QTLs on chromosomes 1, 2, 4, 6, and 12. Moreover, a QTL located in chromosome 7 was identified for low incidence of TP in the RIL population, which was confirmed in ILs with introgressions on chromosome 7. Furthermore, ILs with introgressions in chromosomes 1 and 12 had good FRN and FRS in T3 in replicated trials. These results represent a catalog of QTLs and pre-breeding materials that could be used as the starting point for deciphering the genetic control of the genetic response of reproductive traits at different temperatures and paving the road for developing new cultivars adapted to climate change.

## Introduction

Under the current scenario of global warming, temperature projections estimate a 2–5°C increase in temperature by the end of the twenty-first century (IPCC, 2014). Agriculture production will be greatly affected by this temperature rise, as high temperatures have a negative impact on crops, causing an array of morpho-anatomical, physiological and biochemical changes, which negatively affect plant growth and development and may lead to a drastic reduction in yield (Wahid et al., 2007; Bita and Gerads, 2013). The major losses due to heat stress are expected to occur at low latitude regions (temperate and tropical areas). In fact, yield reduction due to heat stress has been documented in many crops such as wheat, rice, barley, sorghum, maize, chickpea, canola, and more (Hasanuzzaman et al., 2013; Challinor et al., 2014; Alsamir et al., 2019). In the case of tomatoes, a 28% yield reduction due to high temperatures has already been reported (Alsamir et al., 2019).

Tomatoes are one of the most important horticultural crops worldwide and they are currently cultivated in a wide range of agroclimatic regions, either in open fields or under greenhouse conditions. Vegetative growth in tomatoes is well adapted to high temperatures. However, high temperature affects growth and development of different tomato plant organs or structures. For instance, a decrease in the flower number with increased temperatures has been observed (Charles and Harris, 1972) although the effect on fruit set has more dramatic consequences for the yield. Optimal temperatures for setting fruit in field conditions are between 21 and 24°C (Geisenberg and Stewart, 1986) temperatures exceeding 32°C during the day, and/or not decreasing below 21°C during the night (Moore and Thomas, 1952) has dramatic consequences for fruit set and total yield (El Ahmadi and Stevens, 1997). The decrease in tomato fruit set under long-term mildly elevated temperatures has been shown to correlate with a decrease in pollen viability (Dane et al., 1991; Peet et al., 1998; Pressman et al., 2002, 2006; Xu et al., 2017b). An inserted stigma is also an important trait to ensure self-pollination in cultivated tomatoes (Rick and Dempsey, 1969; Chen and Tanksley, 2004). Growth at high temperatures may lead to the protrusion of the style above the anther cone (exsertion, Levy et al., 1978; Sato et al., 2006) negatively affecting flower pollination (Charles and Harris, 1972; Dane et al., 1991; Xu et al., 2017b). High temperatures also increase the incidence of tipburn, necrosis of the apical vegetative and reproductive tissues that have been related to insufficient water absorption and nutritional unbalance (Starck et al., 1994; Chung et al., 2010).

Screening for heat tolerance among tomato cultivars has been carried out in a limited number of cultivars, uncovering just a few “thermotolerant” genotypes (Dane et al., 1991; Opena et al., 1992; Abdul-Baki and Stommel, 1995; Grilli et al., 2007; Kugblenu et al., 2013; Xu et al., 2017b; Ruggieri et al., 2019). Most of the screenings were based on the capacity of plants to set fruit at high temperatures. Given the complexity of this trait, which is also affected by many other factors (Charles and Harris, 1972; Abdul-Baki, 1991; Dane et al., 1991; Adams et al., 2001; Alsamir et al., 2019) some authors have based their screenings on pollen viability as well (Dane et al., 1991; Paupière et al., 2017; Driedekons et al., 2018) as this trait appears to be the most sensitive to high temperatures and under the hypothesis that the dissection of the processes from flower to fruit set is an effective strategy to determine the genetic basis of thermotolerance in tomatoes.

Accessions from wild species of *Solanum* spp. such as *S. pimpinellifolium*, L., *S. pennellii* Correll, *S. habrochaites* S. Knapp and D. M Spooner, *S. chmielewskii* C. M. Rick et al. and *S. cheesmaniae* (L. Riley) Fosberg have been found to be tolerant to high temperatures (Alam et al., 2010; Nahar and Ullah, 2011, 2012; Golam et al., 2012; Paupière et al., 2017; Driedekons et al., 2018). However, our understanding of the genetic control of heat tolerance in tomatoes is still very limited. Up to the present, very few reports have addressed this issue, resulting in very few QTLs involved in heat tolerance identified in tomatoes (Grilli et al., 2007; Lin et al., 2010; Xu et al., 2017b; Ruggieri et al., 2019; Wen et al., 2019). In general, these studies have been performed using simple mapping populations (F_2_), with a limited sample size or with low dense marker coverage. As a consequence, the number of reported QTLs involved in heat tolerance is relatively low and the stability of their effects still needs to be verified.

There are many reasons that may explain the lack of information on the genetic control of heat tolerance in tomatoes. The choice of the proper trait to evaluate this tolerance is a crucial matter. Reproductive traits have been used extensively, including flowering traits, pollen viability and/or fruit set (for example Xu et al., 2017a). On the other hand, the heat injury index and physiological traits have been investigated less frequently (Wen et al., 2019). The relationship between both types of traits has not been studied, although it would be critical for understanding the global response to abiotic stress. Only a tiny fraction of the tomato germplasm has been screened for heat tolerance, more screening efforts would help to identify tolerance sources and to develop mapping populations suitable to investigate the different factors involved in this tolerance. Lastly, the lack of powerful mapping populations such as recombinant inbred lines (RILs), introgression lines (ILs) or multiparent advanced generation intercross (MAGIC) designed for the study of heat tolerance hampers the identification of QTLs.

In this manuscript, we take advantage of the availability of two populations derived from the cross between *S. lycopersicum* cv. ‘Moneymaker’ and *S. pimpinellifolium* accession TO-937: a set of 168 RILs (Alba et al., 2009) and a set of 52 ILs (Barrantes et al., 2014). Even though TO-937 has not been cataloged as a heat tolerant genotype, transgressive segregants are often obtained when crossing cultivated and exotic or wild genotypes (de Vicente and Tanksley, 1993; Diaz et al., 2014). In fact, transgressive QTLs have been identified in the TO-937-x-MoneyMaker RIL and IL populations for other traits previously, for example QTLs involved in disease resistance, vegetative growth and fruit quality (Alba et al., 2009; Powell et al., 2012; Salinas et al., 2013; Capel et al., 2015, 2017; Barrantes et al., 2016). In the current work, a first evaluation of both populations for reproductive traits in different temperature regimens was conducted, finding a promising segregation of heat tolerance in both of them. Therefore, we decided to study them in further replicated experiments to obtain insights on the genetic control of the heat tolerance segregation found in these populations. The joint analysis of those populations could also be a powerful approach for such complex traits. For example, Rambla et al. (2017) successfully studied the genetic control of fruit volatile composition with the joint analysis of both populations. On one hand, RIL genetic architecture is appropriate for the study of traits under complex genetic control and to map QTLs with a relatively good resolution, whereas IL genetic architecture allows the accurate estimation of the effects of single QTL in a desired genetic background, facilitating QTL cloning and integration of QTL in applied marker assisted selection breeding.

This report describes the identification of candidate QTLs involved in the capacity of set fruits under different temperature regimens that can be used to improve heat tolerance in tomatoes.

## Materials and Methods

### Plant Material and Growing Conditions

Two mapping population derived from the cross between *S. lycopersicum* cv. “MoneyMaker” (MM) and the *S. pimpinellifolium* accession TO-937: 167 Recombinant Inbred Lines population (RILs) (Alba et al., 2009) and 56 Introgression Lines (ILs), each one carrying a different introgression from TO-937 into MM genetic background (Barrantes et al., 2014), were investigated in the current research.

Mapping populations were cultivated in greenhouses under controlled temperature conditions in two facilities: Centro de Experiencias Cajamar belonging to Fundación Cajamar Comunidad Valenciana (FCCV, Paiporta, Spain) and National University of Taiwan (NTW, Taipei, Taiwan).

The 167 RILs were assayed for two consecutive years, 2016 and 2017, at FCCV, with three plants per RIL. The three plants were planted in the same bag, thus for statistical analysis, the RIL value was the mean of the three plants. Also, 25 replicates of MM and three of TO-937 were assayed in 2016. In 2017, due to limitations of seed availability for TO-937, only eight replicates of MM were assayed. Plants were grown under a stepwise temperature increase (T1: 25°C day/20°C night; T2: 30°C day/25°C night; T3: 35°C day/30°C night) as follows: each temperature regimen was established for 4 weeks. For 2 weeks, the plants were allowed to flower without any restriction. In the third week, the number of flowers was recorded and in the fourth week the fruit set was recorded as the number of observed developing fruits. After recording the fruit set, all the flowers and fruits were pruned from the plant, in order to avoid the physiological effects of previous fruit load in the new inflorescences, and the temperature was increased to the next regimen.

In addition, the RIL population was also analyzed in the year 2018 in greenhouses under controlled conditions at the National University of Taiwan (NTW). RILs were grown under one of the following temperatures conditions: T2 (30°C day/25°C night), T3 (35°C day/30°C night). The plants were not subjected to stepwise temperature increase; rather they were cultivated in different greenhouses for each temperature treatment.

A preliminary analysis of the 56 ILs was carried out in 2016 at FCCV with the same experimental set up as the RILs, with one replicate of three plants per IL and 25 replicates for MM, evenly distributed among the ILs. A selection of the 12 more promising ILs (SP_1-3, SP_1-4, SP_2-2, SP_5-5, SP_6-3, SP_7-4, SP_11-4, SP_12-1, SP_12-2, SP_12-3, SP_12-4, SP_12-5) were re-evaluated in 2017 at FCCV using the same temperature regimens. In 2019, the experiment was replicated again - three ILs (SP_12-1, SP_12-2 and SP_12-4) with low FRS in 2017 were replaced with ILs with introgressions from chromosome 2 and 7 (SP_2-4, SP_2-5, SP_7-3) as QTLs were previously identified in those genomic regions using the RILs. For all these experiments, ILs were grown following a completely randomized design with five and three replicates (with three plants each) of each genotype in 2017 and 2019, respectively, and 6 replicates of MM with three plants per replicate. Additionally, ILs SP_7-1, SP_7-2, SP_7-3 and SP_7-4, selected to verify tolerance to tipburn (TB, see below), were included in the 2017 experiment at FCCV with a completely randomized design with four replicates of three plants.

### Phenotyping

On the third week of each temperature treatment the number of flowers (FLN), the degree of stigma exsertion (SE, scored as 0: not exserted, 1: slightly exserted and 2: very exserted) were recorded in the second and third truss. The number of fruits set (FRN), and fruit set proportion (FRS = 100 × FRN/FLN) and the incidence of tipburn (TB) on apical tissues was recorded on the fourth week as presence/absence (Table 1).

**TABLE 1:** Description of the reproductive traits studied in the current report.

**Trait name**	**Trait abbreviation**	**Trait description**
Flower number	FLN	Mean of flower number in the second and third truss for each temperature
Fruit number	FRN	Mean of fruit set in the second and third truss for each temperature
Fruit Set Proportion	FRS	100 × (Ratio FRN/FLN)
Stigma Exsertion	SE	0: no exserted, 1: slightly exserted and 2: very exserted
Tipburn	TB	Apical necrosis. 0: no TB and 1: TB incidence
Pollen tube germination	TG	Number of germinated pollen grains
Aniline Blue	AB	Pollen viability based on aniline blue staining
Viable pollen	VP	Percentage of viable pollen based on flow cytometry

Several traits related to pollen viability were measured for the 167 RILs in 2016 and 2017. For pollen tube germination (TG), recorded in 2016 and 2017, pollen was collected directly from fresh flowers and cultivated for 16 h at room temperature in an 18% sucrose medium. TG was measured as the number of germinated grains (scored as 0: no germination, 1: between 1 and 25% germination, 2: between 26 and 75% germination and 3: more than 75% of the pollen grains germinated) counted with a stereomicroscope with epi-illumination. Pollen viability was assessed in 2017 with two techniques: Aniline Blue staining (AB) and flux cytometry of three flowers from each plant. The aniline blue stain was used to identify and evaluate pollen grains by visualization with a Nikon Eclipse E600 microscope, the grain number was scored as 0: no pollen, 1: 25% of the pollen stained, 2: between 26 and 75% stained and 3: more than 75% of the pollen grains stained. The images from the microscope were analyzed with Image J software^1^ to calculate the pollen number (AB) (number of grains in 200 μM). Cytometry analysis was performed in the Enza-Zaden España S.L. facilities with an AmphaTMZ32 flow cytometer (Amphasys AG, Switzerland) to measure the percentage of viable pollen (VP).

### Statistical Analysis

The basic statistics (mean, standard deviation, maximum and minimum values), trait distribution and the Pearson correlations were calculated among traits, years and treatments. IL means were contrasted with the recurrent control MM mean with a Dunnett’s test (*p* < 0.05) in 2017 and 2018 experiments. The analyses were implemented with JMP (2019) software (version 12.1.0).

### QTL Analysis

The map used for the QTL analysis was previously generated and contained 4,932 Single Nucleotide Markers (SNP) from the 8K SNP SOLCAP Infinium chip (Sim et al., 2012). The map was condensed to 1,279 SNPs with QTL IciMapping (Meng et al., 2015) to facilitate the computational analysis. Multi-environmental QTL analysis was performed with IciMapping. LOD threshold for a significance level *p* < 0.05 was obtained by a permutation test with 1,000 resamplings. Additionally, composite interval mapping (CIM, Zeng, 1994) was performed for each independent experiment with Windows QTL Cartographer 2.5^2^ (Wang et al., 2007). Multi-environment QTLs were named with an abbreviation of the trait, followed by the chromosome number, the number of the QTL within chromosome, the temperature regimen (T1, T2 or T3) and the suffix _2E (indicating two environments). QTLs for single experiments were named accordingly, adding a suffix with the experiment year (i.e., _16 and _17). As TP was scored as presence/absence, a χ^2^ test was performed for each marker.

## Results

### Phenotypic Variation for Reproductive Traits at Different Temperatures in the RIL Population

Table 2 depicts the mean for the reproductive traits among the parent genotypes TO-937 and MM in the 2016 and 2017 experiments (TO-937 could not be assayed in 2017). MM maintained flower production in the three temperature regimens, but fructification decreased drastically (between 75 and 85%) at T2 and T3 in both years as a consequence of the heat stress. TO-937 did not set fruit at T2 and T3, or flower at T3, showing even more sensitivity to heat stress.

**TABLE 2:** Means and standard deviation for reproductive traits flower number (FLN), fruit number (FRN) and fruit set proportion (FRS) among the parent genotypes TO-937 and MoneyMaker in the three temperature regimens (T1: 25°C day/20°C night; T2: 30°C day/25°C night; T3: 35°C day/30°C night) from the 2016 and 2017 recombinant inbred line experiments.

	**2016**		**2017**	
	**TO-937**	**MoneyMaker**	**TO-937**	**MoneyMaker**
FLN_T1	12.170.57	8.051.12	nd	8.811.71
FLN_T2	6.831.25	8.222.08	nd	5.881.55
FLN_T3	0	7.1016.03	nd	4.501.45
FRN_T1	10.331.04	5.702.19	nd	8.711.76
FRN_T2	0	0.732.04	nd	1.831.21
FRN_T3	0	1.009.46	nd	0.810.68
FRS_T1	84.9312.86	70.813.54	nd	98.732.4
FRS_T2	0	8.831.99	nd	32.3618.82
FRS_T3	0	14.0820.50	nd	18.1513.39

*nd: no data.*

**FIGURE 1:**
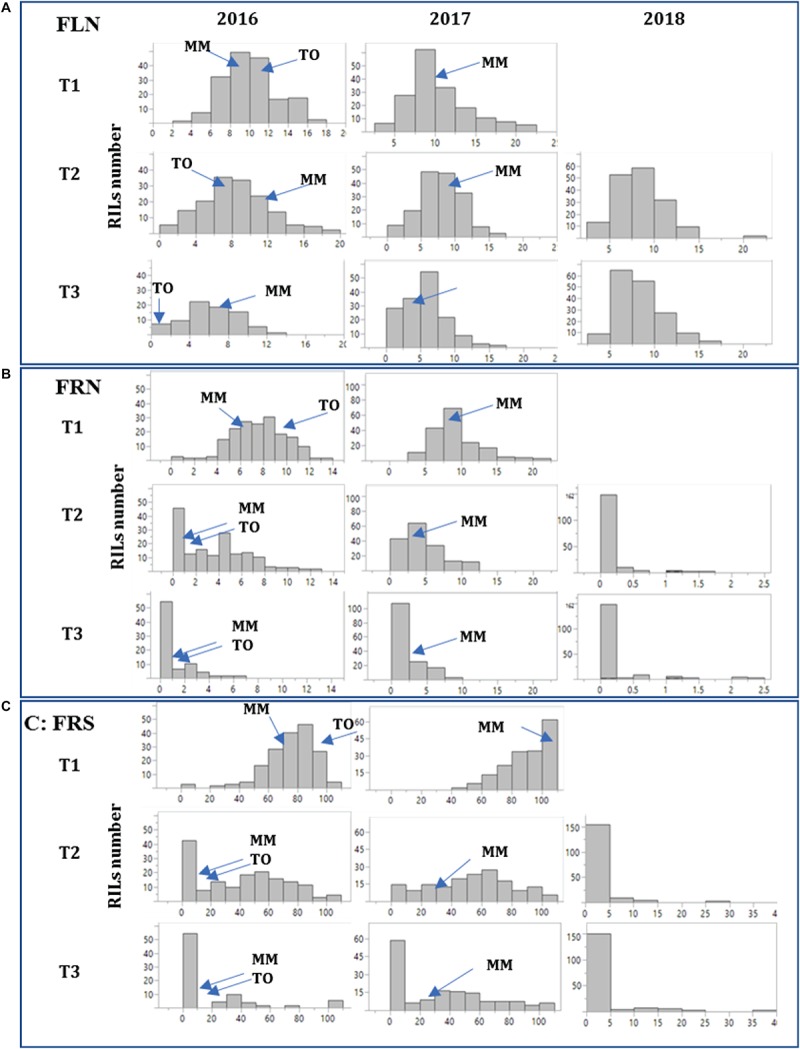
Histograms depicting the distribution of reproductive traits in the RIL population. Reproductive traits flower number (FLN, **A**), number of fruits per inflorescence (FRN, **B**) and percentage of fruit set (FRS, **C**) were studied in three experiments (2016, 2017, and 2018) at three temperature regimens (T1: 25°C day/20°C night; T2: 30°C day/25°C night; T3: 35°C day/30°C night) except in 2018, where the temperature T1 was not assayed. The frequency histograms for each trait in the “MoneyMaker”-x-TO-937 Recombinant Inbred Lines (RIL) across the years are depicted. Means of the parents “Moneymaker” (MM) and TO-937 (TO) are indicated with arrows for the 2016 experiment.

The RIL population MM x-TO-937 was evaluated for reproductive traits at the different temperature regimens in a preliminary experiment in 2016. A transgressive segregation was observed at T2 and T3, with a number of RILs showing a high fruit set at high temperatures, suggesting the presence of genetic variability for heat tolerance in the current RIL population (Figure 1 and Supplementary Table S1). Therefore, RILs were evaluated in two additional experiments (2017, 2018). The distributions of the reproductive traits FLN, FRN and FRS among different experiments and temperature regimens are shown in Figure 1 and Supplementary Table S1. At T1 (optimal temperature), trait distributions generally fitted into a normal distribution. The range of the distributions were wide, transgressive segregants and were observed for all traits in both directions (very low and very high values). However, at T2 the shape of the distributions changed significantly (Figure 1), skewing toward lower values. FRN and FRS dropped more drastically than FLN, although transgressive segregants toward higher values were obtained. At T3, the skew toward low values was even more dramatic with an important proportion of RIL displaying very low values, although a transgressive segregation to higher values was also observed. In the case of FRN and FRS, the median corresponded to values equal or very close to 0. The effect of the themperature increase was even more dramatic in the 2018 experiment performed by NTW. The different facilities used in NTW (a medium sized greenhouse compared with a large greenhouse in the FCCV facilities), external environmental effects (higher humidity in NTW) and the different method used to impose the temperature regimen (stepwise in FCCV vs. direct in NTW) may explain the differences in trait distribution. Nevertheless, these results showed that the reduced ability to produce fruit at high temperature was mostly due to the reduction in the plant’s ability to set fruits rather than due to the reduction in the number of flowers. The broad range of the distributions and the observation of transgressive segregants in different experiments for heat tolerance demonstrated the presence of genetic variability for heat tolerance in this population, highlighting that several RILs were capable of setting fruit even at extremely high temperatures.

The consistency and robustness of the results was tested by correlation analysis for each trait-temperature combination over the different experiments. Correlations among years for the FLN were positive and highly significant at every temperature regimen (Table 3), although correlations were more robust among the 2016 and 2017 experiments than with the 2018 experiment. FRN was also highly correlated between the 2016 and 2017 experiments at the three temperature treatments. Correlations of FRN and FRS between both 2016 and 2017 with 2018 were not significant indicating that non-controlled factors affected that experiment as discussed above. Regarding the correlations between traits within temperature regimens, high correlations were observed between FLN and FRN in 2016 and 2017 experiments at all temperatures. Correlations between FRN and FRS were also generally significant and high in the all experiments and temperatures. On the other hand, FLN and FRS showed variable correlations, but these were mainly non-significant or negative.

**TABLE 3:** Linear correlations among the three temperature treatments (T1: 25°C day/20°C night; T2: 30°C day/25°C night; T3: 35°C day/30°C night) and experiments (2016, 2017, and 2018) for flower number (FLN), fruit number (FRN) and fruit set proportion (FRS) in the MM-xTO-937 recombinant inbred population. Correlations among years are highlighted in yellow.

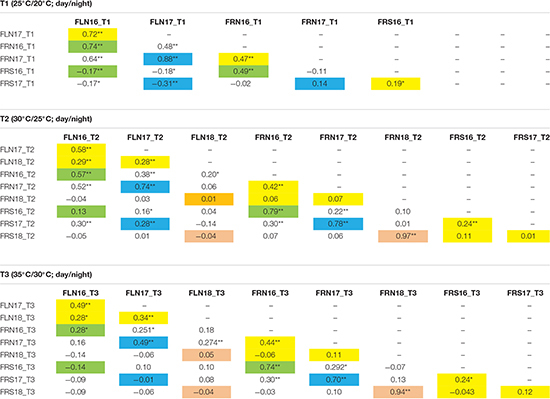

Correlations among traits within experiment are highlighted in green (2016), blue (2017) and orange (2018) at *p < 0.05 and **p < 0.01.

### Identification of QTL Involved in Reproductive Traits in the RIL Population

Due to the low correlation of the 2018 experiment results with 2016 and 2017 data (Table 3), a multi-environment QTL analysis was performed only with 2016 and 2017 data. A total of 22QTL were detected across traits and temperature regimens (Table 4). QTL analysis performed independently in each experiment showed a total of 46 QTLs, 10 of them from the 2018 experiment (Supplementary Table S2) and 34 of them were detected in two experiments or at least in two temperature conditions. QTLs detected in just one experiment or temperature regimen were not considered as reliable QTLs, so they were not taken into account for further discussion. Most of the QTLs detected by multi-environment analysis were also detected by single environment analysis (Table 4 and Supplementary Table S2).

**TABLE 4:** QTLs detected for the reproductive traits flower number (FLN), fruit number (FRN) and fruit set proportion (FRS) and stigma exsertion (SE) at different temperature regimens (T1: 25°C day/20°C night; T2: 30°C day/25°C night; T3: 35°C day/30°C night) in two experiments (2016 and 2017) by multi-environment QTL analysis with ICIMapping.

**Trait**	**Temp.**	**QTL**	**Chr**	**Peak marker**	**position (cM)**	**LOD**	**LOD (A)**	**LOD (AbyE)**	**PVE**	**PVE (A)**	**PVE (AbyE)**	**Add**
FLN	T1	*fln2.1_T1_2E*	2	solcap_snp_sl_35759	39	5.84	4.63	1.21	9.14	5.18	3.96	0.68
		*fln2.2_T1_2E*	2	solcap_snp_sl_20052	108	12.38	5.39	7.00	10.53	6.27	4.26	–0.75
		*fln6.1_T1_2E*	6	solcap_snp_sl_57398	67	6.60	5.49	1.10	6.25	6.20	0.05	0.75
		*fln10.1_T1_2E*	10	solcap_snp_sl_8835	80	7.02	6.90	0.11	10.47	8.09	2.38	0.84
	
	T2	*fln2.2_T2_2E*	2	solcap_snp_sl_36189	105	8.67	7.01	1.66	8.02	7.87	0.15	–1.03
		*fln4.1_T2_2E*	4	solcap_snp_sl_41710	42	4.14	3.42	0.72	3.80	3.77	0.03	0.76
		*fln6.1_T2_2E*	6	solcap_snp_sl_57327	71	4.31	3.06	1.25	3.60	3.37	0.23	0.69
		*fln11.1_T2_2E*	11	solcap_snp_sl_21007	29	5.28	2.64	2.64	3.99	2.93	1.07	–1.01
	
	T3	*fln2.1_T3_2E*	2	solcap_snp_sl_49669	58	5.54	5.54	0.00	9.52	7.09	2.42	0.67
		*fln4.1_T3_2E*	4	solcap_snp_sl_41710	42	4.40	4.40	0.00	7.38	5.51	1.87	0.63

FRN	T1	*frn1.1_T1_2E*	1	SL20127_1427	59	4.79	3.60	1.20	4.71	4.54	0.17	0.53
		*frn2.2_T1_2E*	2	solcap_snp_sl_36189	105	8.16	3.55	4.61	7.01	4.49	2.52	–0.53
		*frn6.1_T1_2E*	6	solcap_snp_sl_41840	44	5.33	3.23	2.10	4.82	4.14	0.68	0.51
		*frn6.2_T1_2E*	6	solcap_snp_sl_57327	72	4.24	4.06	0.18	7.14	5.22	1.92	0.58
		*frn10.1_T1_2E*	10	solcap_snp_sl_59416	65	5.51	4.62	0.89	10.16	6.00	4.16	0.61
	
	T2	*frn4.1_T2_2E*	4	solcap_snp_sl_41710	42	4.64	4.59	0.05	6.15	6.01	0.14	0.70
		*frn6.2_T2_2E*	6	SGN-U579138_snp745	82	4.59	4.21	0.38	6.22	5.52	0.69	0.64
		*frn7.1_T1_2E*	7	solcap_snp_sl_70700	84	6.16	4.70	1.46	8.71	6.22	2.49	–0.67

FRS	T2	*frs6.2_T2_2E*	6	SGN-U579138_snp745	82	4.94	4.65	0.29	7.39	6.83	0.56	6.49
		*frs7.2_T2_2E*	7	solcap_snp_sl_37060	82	7.50	5.38	2.13	11.79	7.91	3.88	–6.85

SE	T1	*se2.1_T1_2E*	2	solcap_snp_sl_50058	79	10.35	9.88	0.47	12.83	12.75	0.08	–0.27
	
	T2	*se2.1_T1_2E*	2	solcap_snp_sl_50058	82	5.63	5.55	0.08	9.51	8.46	1.05	–0.16

*QTLs are named with the trait abbreviation, followed by the chromosome number, the number of QTLs within the chromosome, temperature regimen and the suffix _2E. The marker closest to the maximum LOD score and its genetic position are also shown. The statistical estimates for each QTL include: Maximum LOD score for genetic effects (LOD), LOD score of additive effects [LOD (A)], LOD score for the interaction of additive effects with environment [LOD (AbyE)], percentage of phenotypic variance explained by the QTL (PVE), by the additive effects (A), interaction additive by environment (AbyE) and the additive value (Add), being negative when TO-937 alleles increased the trait and positive when MM increased it.*

A total of five QTLs were detected for FLN by multi-environmental analysis across temperatures (Table 4). Among them, the QTLs with more stable effects among temperatures and years was localized to chromosome 2 in the 105 cM positon (*fln2.1_T1-2E*, *fln2.1_T2_2E*). The phenotypic variance explained by this QTL ranged between 8 and 10% with low QTL-x-environment (QTL-x-E) interaction and with the TO-937 allele increasing FLN. Other QTLs detected in two temperature regimens mapped onto chromosomes 4 and 6 (Table 4). Additionally, a QTL was detected in the 2018 experiment on chromosome 11 at T3 (*fln11.1_T3_18*, Supplementary Table S2), which could correspond to *fln11.2_T2* (Table 4), although they were detected at different temperature regimens. Interestingly, a number of QTLs on chromosomes 2, 4, 6, and 11 showed effects on different temperatures, with MM or TO-937 alleles, depending on the QTL, increasing FLN.

Eight multi-environment QTLs were identified for FRN (Table 4). QTLs on chromosome 4, 6, and 7 were detected at T2, with low QTL-x-E interaction and the TO-937 allele increasing FRN for *frn7.1_T2_*2*E*, whereas the MM allele increased the trait for the other two (*frn6.2_T1_2E* and *frn6.2_T2_2E*). These QTLs were also detected by single environment analysis (Supplementary Table S2). QTLs on chromosome 2 were detected in the three temperature conditions (*frn2.1_T1_16*, *frn2.1_T1_17, frn2.1_T2_16, frn2.1_T2_17*, *frn2.2_T3_17*, Supplementary Table S2) and located in the same region as *frn2.2_T1_2E* (100–130 cM, Table 4). QTLs on chromosome 12 (*frn12.1T2_18* and *frn12.1_T3_18*) were also detected in the 2018 experiment (Supplementary Table S2).

Two multi-environment QTLs for FRS on chromosomes 6 and 7 were mapped in T2, overlapping with FRN QTLs in the same chromosome region (Table 4). As for FRN, the MM allele of *frs6.1_T2_2E* increased FRS, whereas the TO-937 allele increased the trait for *frs7.1_T2_2E. frs6.1_T2_2E* displayed low QTL-x-E interaction, whereas it was more important for *frs7.1_T2_2E*. Additionally, QTLs on chromosome 12 *frs12.1_T2_18* and *frs12.1_T3_18* were detected in the 2018 experiment in the two high temperature regimens, with TO-937 alleles increasing FRS (Supplementary Table S2). Overall, the percentage of variance explained by the QTLs detected by multi-environment analysis or in two temperatures/experiments by single environmental analysis were relatively modest (5–12%, Table 4 and Supplementary Table S2).

One multi-environment QTL for SE was detected on chromosome 2 at T1 and T2 (Table 4). These QTLs explained between 9.5 and 15% of the phenotypic variance and showed low QTL-x-E interaction. QTLs in the same genome region were also detected in the 2018 experiment, with the TO-937 alleles increasing SE (Supplementary Table S2).

### Analysis of Reproductive Traits in Introgression Lines

**FIGURE 2:**
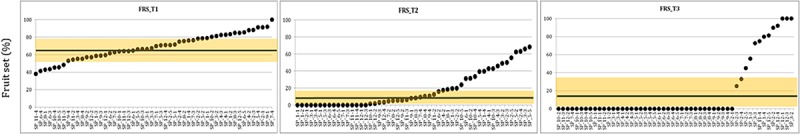
Overview of the fruit set percentage among ILs in 2016 experiment. Means of percentage of fruit set (FRS) in 2016 experiment at three temperature regimens: (T1: 25°C day/20°C night; T2: 30°C day/25°C night; T3: 35°C day/30°C night) among TO-937 introgression lines (ILs). ILs are on the X axis in order according their FRS value. The yellow line indicates the “MoneyMaker” mean and the shadowed area the standard deviation.

The complete set of 56 ILs was analyzed for the 2016 experiment. At T1 and T2, the FLN means were similar in MM and IL population for both temperatures, whereas FLN clearly decreased at T3 (Supplementary Table S3 and Figure S1). In the case of FRN and FRS, a drastic decrease was already observed at T2 (Supplementary Table S3 and Figure S1). Mean differences between ILs and MM were not important for FLN at T1 and T2, and no IL overcame MM at T3 (Supplementary Table S3). Regarding FRN, ILs with higher FRN than MM were observed at T2 and T3 (Supplementary Table S3), suggesting that some ILs may carry heat tolerance genes. Lastly, few ILs displayed higher FRS than MM at T1, 19 ILs at T2 and 12 ILs at T3 (Figure 2). Interestingly, ILs with introgression in chromosomes 1, 2, and 12 showed high FRS, overlapping with some of the reproductive trait QTLs described above in the RIL population.

In order to verify the heat tolerance of candidate ILs, an assay with five replicates per IL was carried out in 2017. Twelve ILs with better performance under high temperatures in the previous year’s assay were analyzed at the same three temperatures regimens. No significant differences were found between MM and ILs for FLN at all three temperatures (Supplementary Table S3). The same result was found for FRN and FRS at T2 (Supplementary Table S3). At T3, SP_12-5 displayed higher FRN and FRS than MM, whereas SP_1-4 showed higher FRS (Supplementary Table S3). In 2019, a similar experiment was implemented with 9 of the 12 previously selected ILs and three additional ones selected for carrying an introgression in the same position of a QTL detected in the RIL population. SP_1-4 and SP_12-2 showed higher FRN and FRS than MM at T3 (Supplementary Table S3). SP_1-4 also showed a high FRS at T3 in all three experiments. Thus, the QTLs on chromosome 12, *frn12.1_T3_18* and *frs12.1_T3_18* were confirmed with SP_12-2. On the other hand, QTLs for FRN on chromosome 1 were detected in the RILs at T1 (*frn.1_T1_2E*) and T2 (*frn1.1_T2_16*), although not at T3, neither for FRS, whereas SP1-4 showed effects on FRN and FRS at T3.

### Pollen Viability

**FIGURE 3:**
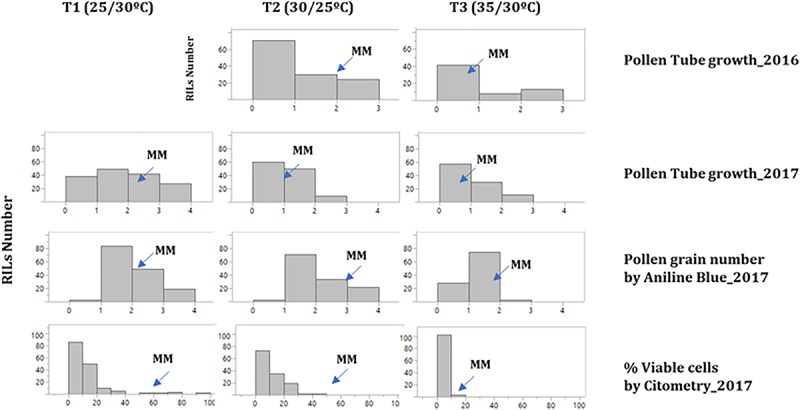
Histograms of the distribution of pollen viability traits in the RIL population. Histograms at the three temperature regimens: (T1: 25°C day/20°C night; T2: 30°C day/25°C night; T3: 35°C day/30°C night) for the pollen viability traits studied in 2016 and 2017 experiments among MoneyMaker”-x-TO-937 Recombinant Inbred Lines (RIL). The mean for “Moneymaker” (MM) is indicated with an arrow. In 2016, pollen tube growth was not assayed at T1.

Pollen viability was determined by three different approaches: pollen tube germination (TG), aniline blue staining (AB) and cytometry (VP) for the 2017 experiment (at all three temperatures), whereas in 2016 only TG was analyzed at T2 and T3. The distribution of the traits showed a decrease of all pollen viability traits as temperature increased, mostly in T3 (Figure 3). A low correlation was found for TG between 2016 and 2017 (Supplementary Table S4). On the other hand, correlations between AB and VP were positive and highly significant at all three temperature regimens (Supplementary Table S4). However, TG did not show a significant correlation with the previous traits. Regarding to the correlation between pollen and the other reproductive traits, no significant correlations were found between pollen viability traits and FLN, FRN or FRS (Supplementary Table S4).

In 2016, no QTL was identified for TG. In 2017, 4 QTLs for TG, 2 QTLs for AB and 2 QTLs for VP were identified (Table 5). A region at the end of chromosome 3 contained QTLs involved in all three pollen viability traits at T1, with the TO-937 allele increasing viability, which could reflect the presence of a QTL involved in pollen viability at normal temperatures. Interestingly, the QTL for TG and AB were detected at the top of chromosome 7 at T2, which could be a candidate for a pollen viability QTL under mild heat stress. Nevertheless, as these QTLs were studied in only one experiment, their effects should be verified with additional experiments.

**TABLE 5:** QTLs detected for the pollen viability traits pollen tube growth (TG), aniline blue staining (AB) and viable pollen by cytometry (VP) at different temperature regimens (T1: 25°C day/20°C night; T2: 30°C day/25°C night; T3: 35°C day/30°C night) in the 2017 experiment with the Moneymaker-x-TO-937 recombinant inbred lines.

**Trait**	**Temp.**	**QTL**	**Chr**	**Peak marker**	**position (cM)**	**LOD**	**(%) Phenotipic variance**	**a**
TG	T1	**TG3.1_T1**	3	solcap_snp_sl_20802	120.0	3.3	7.49	–0.30
		TG8.1_T1	8	solcap_snp_sl_64604	88.07	3.6	5.86	0.27
	
	T2	TG7.1_T2	7	solcap_snp_sl_62904	1.0	3.8	9.15	–0.23
	
	T3	**TG3.1_T3**	3	solcap_snp_sl_61460	134.32	4.4	1.85	0.14

AB	T1	AB3.1_T1	3	CL016802-0675	95.0	3.1	9.76	–0.28
	
	T2	AB7.1_T2	7	solcap_snp_sl_62904	1.0	2.9	6.42	–0.26

VP	T1	VP3.1_T1	3	solcap_snp_sl_58584	92.14	3.1	9.77	–4.29
		VP4.1_T1	4	solcap_snp_sl_47457	122.32	29.5	61.52	32.41

*QTLs are named with the trait abbreviation, followed by the chromosome number, the number of QTLs within the chromosome and the temperature regimen. The additive value (A) is negative when TO-937 alleles increase the trait and positive when “MoneyMarker” alleles increase it. QTLs detected in at least two temperatures or experiments are highlighted in bold.*

### Tipburn Incidence

**FIGURE 4:**
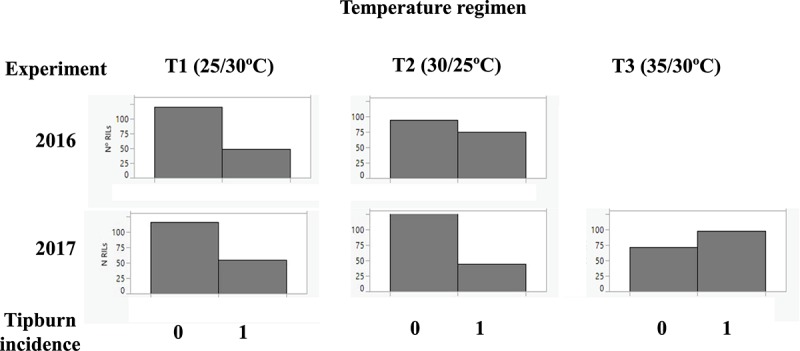
Histograms of incidence of tipburn. Tipburn incidence was scored as presence (1) and absence (0) at the three temperature regimens: (T1: 25°C day/20°C night; T2: 30°C day/25°C night; T3: 35°C day/30°C night) in the 2016 and 2017 experiments. The frequency of tipburn incidence among “MoneyMaker”-x-TO-937 Recombinant Inbred Lines (RIL) across years and experiments is depicted.

Tipburn is a physiological disorder that usually occurs as a consequence of heat stress (Jenni et al., 2013) and it is characterized by the necrosis of the youngest leaves and inflorescences at the tip of the apex of the plant, bringing production of new vegetative and reproductive structures to a halt. A segregation of tipburn incidence (TB) was observed initially in the RIL population during the 2016 experiment, and it was also recorded in the 2017 experiment. The incidence of TB depended on the temperature regimen: at optimal temperatures TB was present in less than one-third of the plants, while at T3, over half of the RILs showed TB (Figure 4).

**FIGURE 5:**
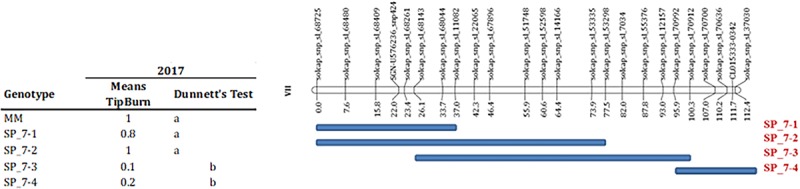
Validation of QTL for tipburn incidence on chromosome 7. The condensed genetic map with SOLCAP markers calculated in the MoneyMaker”-x-TO-937 Recombinant Inbred Lines (RIL) is depicted in the top of the figure. The extent of the TO-937 introgressions for the selected introgression lines (ILs) is indicated with a blue bar below the genetic map. The table on the left depicts the tipburn means for the ILs and “Moneymaker” (MM) in the 2017 experiment. Dunnet’s test column indicates the statistically significant mean differences at *p* < 0.05, means with the different letters indicates significant mean differences.

Several markers showed an association with TP incidence at chromosome 7 at T1 in 2016 and at T3 in 2017 experiments. The marker with the strongest association was located in position 58 cM (Table 6), although a large region of chromosome 7 also showed association with TP (Supplementary Figure S2), what could indicate the presence of a linked QTL. In order to verify this QTL, the incidence of TB was evaluated in ILs with introgressions in chromosome 7. ILs SP_7-3 and SP_7-4 did not show incidence of TP (Figure 5). The introgressions carried by these ILs only overlapped in the interval between markers solcap_snp_sl_70992 and solcap_snp_sl_70912, so it is reasonable to suggest that the QTL is within that marker interval. Nevertheless, the possibility of two linked genes located in the non-overlapping regions of the two ILs cannot be ruled out, and further fine mapping would be necessary to discern between these two hypotheses. SNP genotype data and genetic for RILs can be found in Supplementary Table S5. RIL and IL phenotypic data for all experiments are included in Supplementary Tables S6, S7.

**TABLE 6:** Incidence of Tipburn (TB) among recombinant inbred lines (RILs) in the 2016 experiment at T1 (25°C day/20°C night) (16T1) and the 2017 experiment at T3 (35°C day/30°C night) (T3) and association with marker solcap_snp_sl_53335 (located in position 58 cM of chromosome 7).

**Genotype**	**Number of RILs**	**Experiment**			
		**16T1**		**17T3**	
		**RILs with TB**	**RILs No TB**	**RILs with TB**	**RILs No TB**
EE	58	28	30	42	16
PP	78	57	21	44	34
	*p*	0.0002		0.0011	

*Genotype indicates homozygous “MoneyMarker” (MM) and homozygous TO-937 (PP) for the marker, followed by the total number of RILs with the genotypes and number of RILs within each genotypic class displaying or not displaying TB. In the lower part of the table, the significance of the χ^2^ test for marker/TB association is shown.*

## Discussion

Heat stress affects both vegetative growth and reproduction of plants, and in both cases, the plant response is complex and controlled by multiple genes. The choice of the specific vegetative or reproductive trait for studying heat tolerance will determine the genetic mechanism that can be identified. The genetic mechanism may be common for vegetative and reproductive traits or specific, i. e., depending on the trait a component of the tolerance would be studied. The effects of heat stress on vegetative traits are evident at high temperatures (i.e., 40°C, Wen et al., 2019), whereas reproductive traits are already affected by mild heat stress when minimum temperatures are above 25°C (Xu et al., 2017b). Currently, night temperatures over 25°C are not unusual during summer at regions where tomatoes are cultivated, such as the Mediterranean basin, so we decided to focus our research on identifying sources of heat tolerance on reproductive traits when temperatures increase above the minimum of 25°C.

Reproductive traits can be measured in different ways including the number of flowers, fruits per inflorescence and pollen viability. The last one has been proposed as an adequate indicator of heat tolerance due to its correlation with fruit set and a likely simpler genetic control mechanism (Xu et al., 2017b; Driedekons et al., 2018). In the current report, we have evaluated pollen viability using three different methods: TG, AB and VP. AB and VP showed strong correlations at all temperatures, but correlations with TG were not significant. On the other hand, QTLs involved in TG, AB and VP were detected in the same region of chromosome 3 at T1 with the TO-937 allele increasing pollen viability, although the effects of the QTLs were relatively modest (explaining less than 10% of phenotypic variance). In this same region in chromosome 3, a MetaQTL for pollen viability was detected from the joint analysis of four different experiments (Ayenan et al., 2019). Similarly, QTLs in the same region of chromosome 7 were found for TG and AB in T2, also with modest effect (also less than 10% of phenotypic variance). These results suggested that, even though TG is in general controlled by a different mechanism than AB and VP, some common mechanisms may exist. Xu et al. (2017b) detected a QTL for pollen viability on chromosome 11, but we did not find QTLs for any of the assayed pollen viability traits in that chromosome, which can be explained by the different germplasms used in the two reports. We also did not find correlation between pollen viability traits and fruit set, although the lack of biological replications in the RIL population might limit conclusions from this observation. Xu et al. (2017a) found a significant correlation at high temperature, but this was not significant at the control temperature, suggesting that the correlation may be dependent on the experimental conditions. Given the low correlation between pollen viability traits and the modest effects of the QTLs detected in the current report, the genetic variability for pollen variability traits in the current RIL seems to be low and likely not sufficient for accurately studying their genetic control.

The high and positive correlation observed in the reproductive traits FLN, FRN, and FRS between the 2016 and 2017 experiments indicated a significant hereditability at all the assayed temperatures. The low correlation observed with the 2018 experiment may be due to the environmental differences between NTW and FCCV greenhouse facilities and/or developmental stages of the plants when they were subjected to high temperatures in the NTW facilities (Charles and Harris, 1972). The limitation of biological replicates does not allow us to infer sturdy conclusions from these observations. Also, several QTLs and ILs displayed consistent effects at different temperature regimens and across years, indicating that the current mapping populations harbored sufficient genetic variability for studying the genetic control of these reproductive traits. Xu et al. (2017a) did not find a correlation between pollen viability and female fertility, so it is likely that the fruit set variability in the current experiment could be related to female fertility instead of pollen variability, which would explain the lack of correlation between pollen viability and fruit set.

Therefore, the discussion will be focused on FLN, FRN, and FRS. At mild heat stress (T2), FLN only showed a slight reduction, with this being more drastic at T3. On the other hand, detrimental effects of increased temperature were apparent for mild heat stress on FRN and FRS. Thus, even though, MM and TO-937 showed a drastic reduction of FRN and FRS at T2, and more drastic at T3, transgressive segregations were observed in the RIL population. The transgressive phenotypes appeared due to allelic combinations from the different parents, with a portion of RILs setting fruit at both high temperatures. Transgressive segregation is commonly reported when crossing exotic germplasms with cultivated tomatoes (de Vicente and Tanksley, 1993; Monforte et al., 2001; Shivaprasad et al., 2012; Capel et al., 2015). The fact that transgressive segregation for heat tolerance in reproductive traits has been found in a recombinant population derived from non-heat-tolerant parents reinforces the power of interspecific crosses for uncovering hidden genetic diversity. In some cases both TO-937 and MM alleles are able to increase reproductive traits at a high temperature in different QTLs. Xu et al. (2017a) in a cross between the heat-tolerant cultivar “Nagcarlang” and heat-susceptible NCHS-1, and Wen et al. (2019) in a cross between the heat-tolerant LA2093 (*S. pimpinellifolium*) and heat-susceptible LA1698 (cultivated tomato), also found that alleles from both parents were associated to tolerance. Thus, the tolerance determined in parents does not seem to be a perfect indicator of the genetic potential of the alleles carried by them, which increases the potential of the tomato germplasm for developing new heat tolerant cultivars by generating new genetic combinations.

The number of QTLs detected at each temperature regime/experiment combination was relatively low. Likely, a large number of biological replication could help to detect a larger number of QTLs. This low QTL detection was also observed in the previous works by Xu et al. (2017a); Ruggieri et al. (2019) and Wen et al. (2019). In fact, the meta-QTL analysis carried out by Ayenan et al. (2019) only found one meta-QTL in chromosome 1 for the number of flowers per inflorescence (Ayenan et al., 2019). The low QTL detection probably reflects the genetic complexity of the studied heat tolerance traits, resulting in only a fraction of the genes involved being detected by using common QTL mapping approaches. In the current report, the QTLs displaying more consistent effects across temperatures and experiments were involved in FLN and FRN. Among them, the QTL with the most consistent effect is located on the distal region of chromosome 2. Interestingly, the association of fruit numbers at high temperature with makers located in chromosome 2 was also reported by Ruggieri et al. (2019). Another region which can be highlighted is the FLN QTL on chromosome 11 that may correspond with the QTL qFP11 involved in flowers per inflorescence as reported by Xu et al. (2017a). While it is true that the number of QTL experiments dissecting the genetic control of heat tolerance in tomatoes is still very limited, the identification of similar genomic regions involved in heat tolerance in this and previous reports is encouraging and provides additional proof that we are on the right track.

The analysis of the IL population was initially intended to verify the QTLs detected in the RIL population, but also to identify new ones. Our results show that there was not a very high correspondence between the QTLs identified in both populations. The lack of verification may be due to the complex genetic control of the traits, i.e., the effect of the QTL may greatly depend on the genetic background, so specific multi-loci combinations would be necessary to express the heat tolerance. These multi-loci combinations may occur in a number of RILs, but they disappeared in ILs. Nevertheless, some interesting results were found among ILs. For instance, IL SP12-2 showed higher FRN and FRS at T3 than MM in the 2019 experiment which could indicate the effects of QTLs *frn12.1_T3_18*, *frs12.1_T3_18* detected in the RIL population. SP_1-4 displayed higher FRN and FRS than MM at T3, whereas the QTLs detected in this region with the RILs were involved only in FRN at T1 and T2 temperatures (*frn11.1_T1_16*, *frn1.1_T2_16*). It is likely that the QTL for FRN has stronger effects in the RILs at T1 and T2, whereas in the ILs the effects become more evident at T3 due to the differences in interactions with the genetic background as a result of the different genetic structure of both populations. Nevertheless, we cannot rule out that those are different QTLs. The changes in the genetic background during IL development can also induce new phenotypes that were not observed previously in early segregating populations. Unexpected phenotypes are commonly reported in IL populations, such as the increased intensity of the internal red color in tomatoes observed in a green-fruited *S. habrochaites* IL (Monforte et al., 2001) increased fruit weight from *S. pimpinellifolium* (Barrantes et al., 2016) fruits of a melon IL showing climacteric ripening from non-climacteric parents (Vegas et al., 2013) or production of round fruits from parents that produce oval or elongated fruits (Diaz et al., 2014). As each IL carries a single introgression on the otherwise MM genetic background, the genetic complexity of the traits was reduced to a single locus, which will facilitate their future use in breeding programs and at the same time to identify the causal genes of the heat tolerance.

In addition to the reproductive traits, heat stress affects other factors such as stigma exsertion, increasing the protrusion of the style out of the anther. QTLs with strong effects on SE across experiment and temperature regimens were located on chromosome 2 in the same region as *se2.1* that was map-based cloned by Chen and Tanksley (2004). Xu et al. (2017a) also detected SE QTLs in that region under heat stress, supporting the finding that *se2.1* control of stigma exsertion is mostly independent on temperature conditions. The non-significant correlation between SE and FRS also reinforces the idea that SE is not an appropriate trait for evaluating the tolerance to high temperature in tomatoes (Lohar and Peat, 1997).

Lastly, TB has been studied thoroughly in leafy vegetables (such as lettuce) as the presence of this physiological disorder has a direct impact on the market value. In the case of tomato, the incidence of TB affects young developing tips and inflorescences, dramatically reducing tomato yield. We report a consistent QTL for TB on chromosome 7 successfully validated in ILs SP_7-3 and SP_7-4. According to the RIL experiment, the QTL would map in the central region of the chromosome (around 58 cM), whereas the IL analysis suggested that the QTL would map on the distal regions (around 95 cM), assuming that the QTL is located in the region where the SP_7-3 and SP_7-4 introgressions overlap. A closer look to the association of markers with TB incidence across chromosome 7 showed that markers located in the region between 50 and 100 cM were also associated with TB tolerance (Supplementary Figure S2), which may be interpreted as the presence of multiple linked QTLs. Therefore, we cannot rule out that SP_7-3 and SP_7-4 tolerance to TB is due to the same QTL or different linked QTLs on chromosome 7. Anyway, both ILs showed a very mild, negligible incidence of TB, whereas other ILs and MM were severely affected. This result suggested that TB can be prevented with just one locus (i. e., even though the ILs may carry different QTLs, the presence of only one of them is sufficient for tolerance). To the best of our knowledge, this is the first report of the mapping of TB resistance in tomatoes. Major QTLs for TB have also been recently reported in lettuce (Jenni et al., 2013; Macias Gonzalez et al., 2019) however, no candidate gene has been suggested yet. The precise location of QTL or QTLs on chromosome 7 will allow an assessment of whether the genetic control on TB resistance could be similar in both species, as soon as candidate genes can be defined in posterior research studies.

## Conclusions

The genetic control of heat stress tolerance in tomatoes is largely unknown. In our experimental design, no true biological replicates were studied in the RIL population, which limited some of the conclusions about the general genetic control of heat tolerance in the RIL population. Nevertheless, as geneticists, our interest was focused in the cosegregation of markers and traits to identify genomic regions associated with the tolerance. We identified new sources of variability for heat tolerance and dissected the tolerance to heat stress for reproductive traits using previously developed mapping populations. The strategy was successful, as heat tolerance associated RILs, ILs and markers were identified. The heat tolerance was mainly associated with female fertility rather than pollen viability, although some QTLs involved in pollen viability were also found. Some ILs showed tolerance in several replicated experiments, making them the proper choice for increasing the heat tolerance of tomato cultivars or to define candidate or casual genes. Some QTLs detected in the RIL population could not be verified with the ILs, indicating that the effects of these QTLs are dependent on the genetic background. For those cases, a single QTL selection approach (as the IL strategy) would not be the right approach and other strategies such as genomic selection may be more appropriate (Voss-Fels et al., 2019) for managing them in breeding programs. The study of two mapping populations with different genetic structures has allowed us to obtain some insights into different heat tolerance mechanisms. Also, for the first time, a locus for TB tolerance has been mapped on chromosome 7, and its transfer to applied or basic research will be straightforward. In summary, we report a large catalog of QTLs involved in tomato reproductive traits at different temperatures. Most of these QTLs are not involved in pollen viability traits, but they increase the fruit set at high temperatures. Therefore, it would be expected that in combination with the pollen viability QTL from other sources this could speed up the development of heat tolerant tomato cultivars. Nevertheless, fruit set is one of the components of global heat tolerance. Research on other traits such as vegetative development, fruit quality and postharvest behavior at high temperatures would complement the current study to design a holistic strategy to develop heat tolerant cultivars.

## Data Availability Statement

SNP genotype data for RILs is depicted in Supplementary Table S5. RIL and IL phenotypic data for all experiments are included in Supplementary Tables S6 and S7. All data are also deposited in 10.5281/zenodo.3567014.

## Author Contributions

MG carried out the experiments, analyzed the data, and drafted the manuscript. Y-CL, K-YC, DG, and TM carried out experiments. IN and CB supervised greenhouse experiments. AG and AM designed the study and wrote the manuscript.

## Conflict of Interest

The authors declare that the research was conducted in the absence of any commercial or financial relationships that could be construed as a potential conflict of interest.

The reviewer GR declared a past co-authorship with several of the authors AM and MG to the handling Editor.

## Footnotes

1 https://imagej.nih.gov/ij/

2 http://statgen.ncsu.edu/qtlcart/WQTLCart.htm
