# Supplementary material for: Genetic Control of Reproductive Traits in Tomatoes Under High Temperature
Source: Front Plant Sci. 2020 Apr 24;11:326. doi: 10.3389/fpls.2020.00326 (PMC7193983; doi:10.3389/fpls.2020.00326)
Supplement: Supplementary file 8 [file Presentation_1.PPTX]

## Slide 1
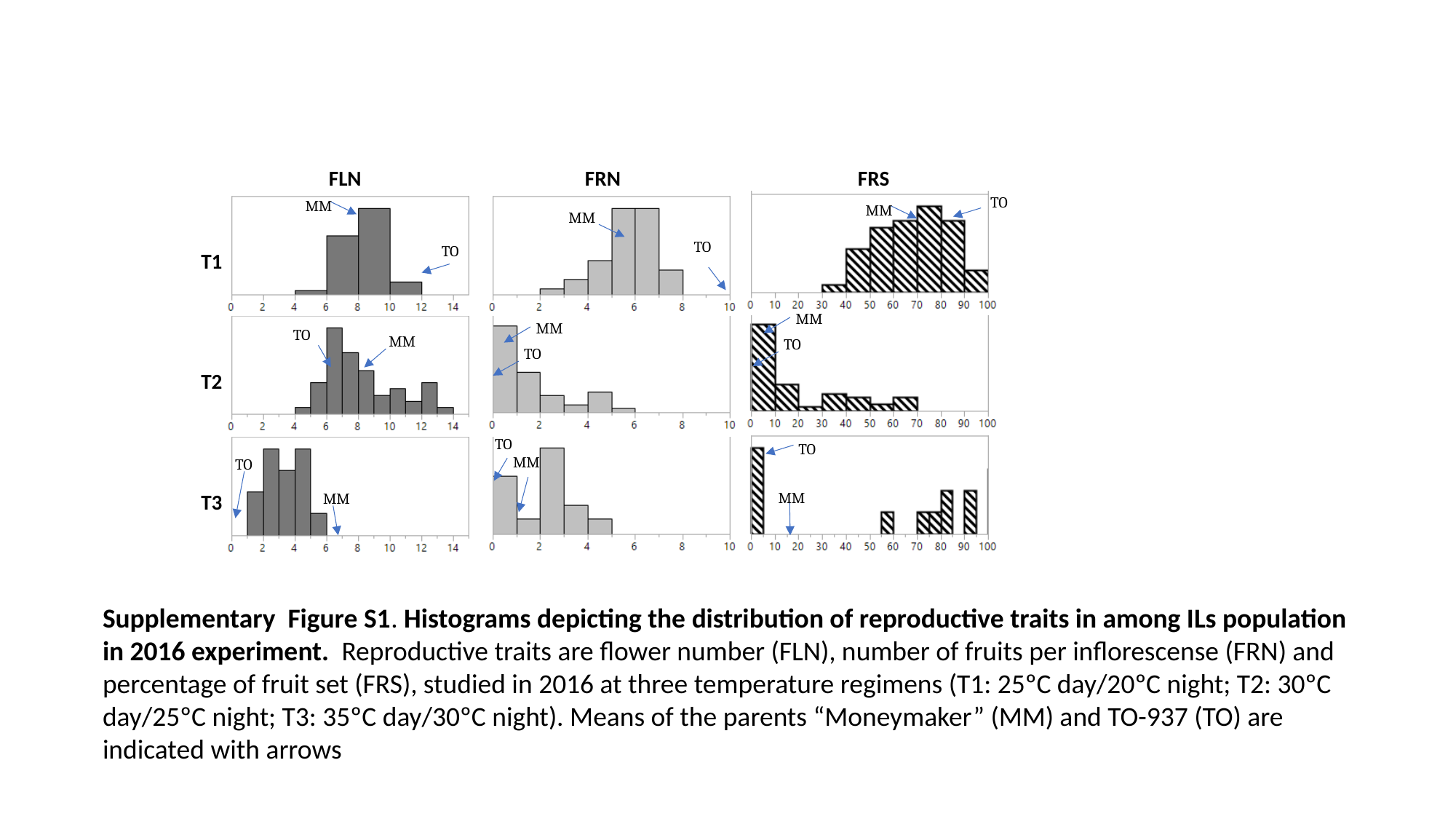

FLN
FRN
FRS
TO
MM
MM
MM
TO
TO
T1
MM
MM
TO
MM
TO
TO
T2
TO
TO
MM
TO
MM
T3
MM
Supplementary Figure S1. Histograms depicting the distribution of reproductive traits in among ILs population in 2016 experiment. Reproductive traits are flower number (FLN), number of fruits per inflorescense (FRN) and percentage of fruit set (FRS), studied in 2016 at three temperature regimens (T1: 25ºC day/20ºC night; T2: 30ºC day/25ºC night; T3: 35ºC day/30ºC night). Means of the parents “Moneymaker” (MM) and TO-937 (TO) are indicated with arrows
