# Supplementary material for: Genetic Control of Reproductive Traits in Tomatoes Under High Temperature
Source: Front Plant Sci. 2020 Apr 24;11:326. doi: 10.3389/fpls.2020.00326 (PMC7193983; doi:10.3389/fpls.2020.00326)
Supplement: Supplementary file 9 [file Presentation_2.PPTX]

## Slide 1
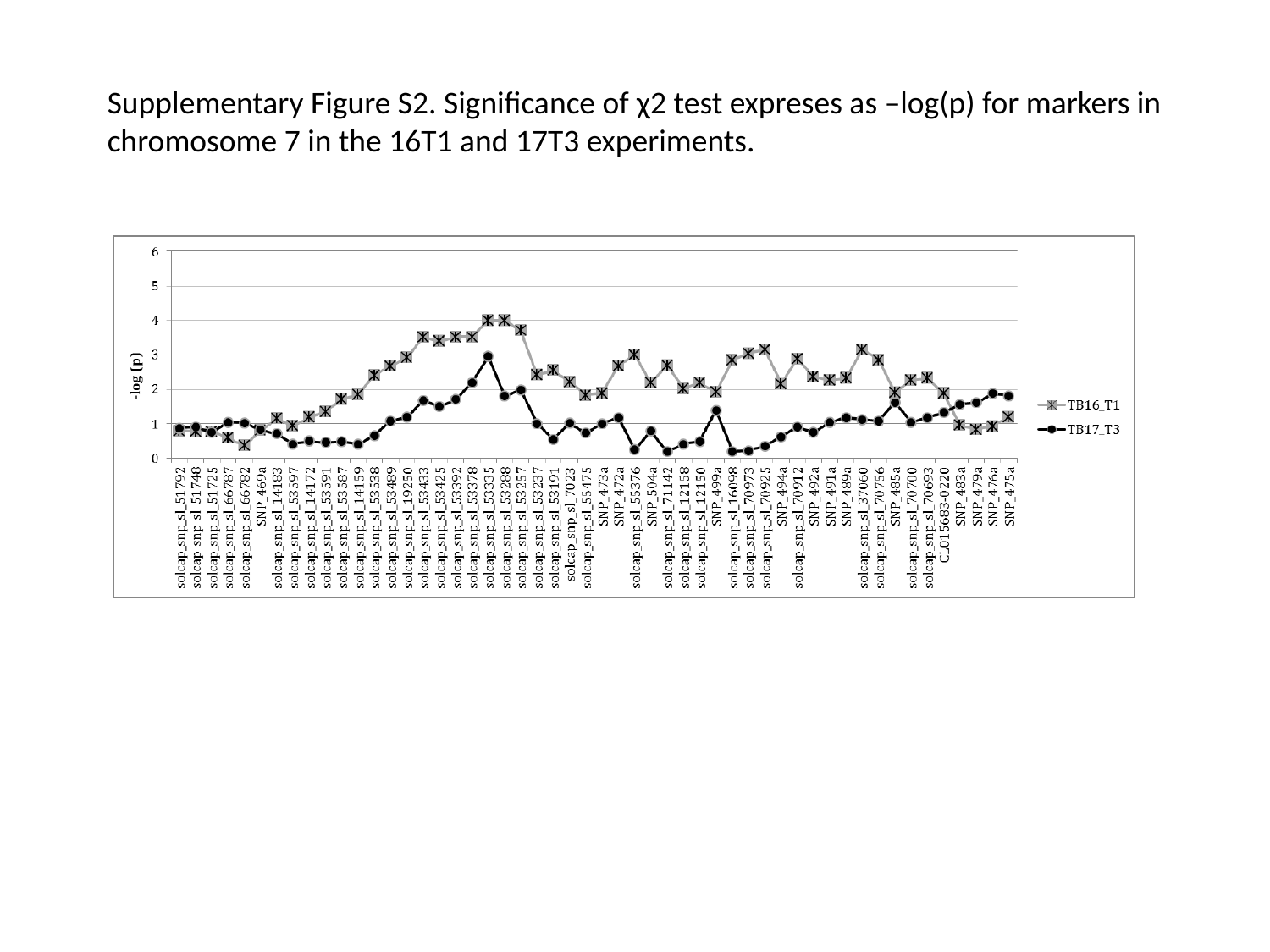

Supplementary Figure S2. Significance of χ2 test expreses as –log(p) for markers in chromosome 7 in the 16T1 and 17T3 experiments.
